# Alpha-2-Macroglobulin Is Acutely Sensitive to Freezing and Lyophilization: Implications for Structural and Functional Studies

**DOI:** 10.1371/journal.pone.0130036

**Published:** 2015-06-23

**Authors:** Amy R. Wyatt, Janet R. Kumita, Natalie E. Farrawell, Christopher M. Dobson, Mark R. Wilson

**Affiliations:** 1 Illawarra Health and Medical Research Institute and School of Biological Sciences, University of Wollongong, Wollongong, NSW 2522, Australia; 2 Department of Chemistry, University of Cambridge, Cambridge CB2 1EW, United Kingdom; Kermanshah University of Medical Sciences, ISLAMIC REPUBLIC OF IRAN

## Abstract

Alpha-2-macroglobulin is an abundant secreted protein that is of particular interest because of its diverse ligand binding profile and multifunctional nature, which includes roles as a protease inhibitor and as a molecular chaperone. The activities of alpha-2-macroglobulin are typically dependent on whether its conformation is native or transformed (i.e. adopts a more compact conformation after interactions with proteases or small nucleophiles), and are also influenced by dissociation of the native alpha-2-macroglobulin tetramer into stable dimers. Alpha-2-macroglobulin is predominately present as the native tetramer *in vivo*; once purified from human blood plasma, however, alpha-2-macroglobulin can undergo a number of conformational changes during storage, including transformation, aggregation or dissociation. We demonstrate that, particularly in the presence of sodium chloride or amine containing compounds, freezing and/or lyophilization of alpha-2-macroglobulin induces conformational changes with functional consequences. These conformational changes in alpha-2-macroglobulin are not always detected by standard native polyacrylamide gel electrophoresis, but can be measured using bisANS fluorescence assays. Increased surface hydrophobicity of alpha-2-macroglobulin, as assessed by bisANS fluorescence measurements, is accompanied by (i) reduced trypsin binding activity, (ii) increased chaperone activity, and (iii) increased binding to the surfaces of SH-SY5Y neurons, in part, via lipoprotein receptors. We show that sucrose (but not glycine) effectively protects native alpha-2-macroglobulin from denaturation during freezing and/or lyophilization, thereby providing a reproducible method for the handling and long-term storage of this protein.

## Introduction

Alpha-2-macroglobulin (α_2_M) is an ancient component of the innate immune system that is highly conserved in animal species separated by over half a billion years of evolution. The best known function of α_2_M is its ability to trap covalently a broad spectrum of proteases and facilitate their clearance via interaction with the low-density lipoprotein receptor-related protein (LRP) [1–3]. α_2_M has also been shown to facilitate the clearance of a diverse range of non-covalently bound ligands including the Alzheimer’s disease-associated amyloid β-peptide (Aβ) [4, 5] and many cytokines and growth factors [6–10]. The binding of α_2_M to heat-denatured and amyloidogenic peptides and proteins inhibits their aggregation and α_2_M is found co-localized with misfolded proteins in disease states [11–17]. Thus, α_2_M is proposed to have important roles in extracellular proteostasis, immune system regulation and tissue remodeling, and has been the subject of over 5000 scholarly articles and reviews since the 1970s [18].

Human α_2_M is a 720 kDa homotetramer comprised of four 180 kDa subunits. These subunits are paired by disulfide bonds to form covalently-linked dimers, which non-covalently associate to complete the cage-like quaternary structure of α_2_M [19]. Protease trapping by α_2_M involves three major steps, namely (i) cleavage of α_2_M in the ‘bait region’ which contains a large number of different protease cleavage sites [20], (ii) covalent cross-linking of the protease to α_2_M via a reactive thioester bond [21], and (iii) a dramatic conformational change which causes α_2_M to become more compact and reveals a cryptic binding site on α_2_M for LRP [2]. In the absence of proteases, small nucleophilic molecules such as methylamine cause α_2_M to adopt a structurally similar compact form by reacting with the thioester bond [2, 22]. Several alternative naming systems have been used to describe the two main conformational states of α_2_M. These include native and transformed [23, 24]; slow and fast, due to the enhanced mobility of the compact form of α_2_M as assessed by native gel electrophoresis [2, 20]; native and activated [5, 16, 25] and active (or functional) and inactivated [26, 27]. The latter two naming systems can cause confusion given that in the compact state α_2_M may be considered “activated” in reference to its newly acquired ability to bind to LRP [5] or α_2_M may be considered “inactivated” in terms of its ability to trap proteases [26, 27]. The term α_2_M-protease complex is also used to describe α_2_M after proteases become trapped within the α_2_M cage [28]. For the purpose of this report the compact conformation of α_2_M will be distinguished from the native state using the term transformed.

The activities of α_2_M are typically dependent on whether its conformation is native or transformed, and may also be influenced by dissociation of the native tetramer into stable dimers (Table 1). In biological fluids native α_2_M is far more abundant than transformed α_2_M, as the latter is cleared very rapidly via LRP [26, 29, 30]. The physiological relevance of human α_2_M dimers has yet to be established; it has been demonstrated, however, that human α_2_M readily forms dimers after exposure to hypochlorite, a potent oxidant produced by activated immune cells [31, 32]. This finding supports the idea that α_2_M dimers may have specialized importance during inflammation. It has been observed that during cold storage purified α_2_M can adopt transformed or partially transformed conformations, dissociate into dimers or form high molecular weight aggregates [33–38]. Despite such observations, to our knowledge, no previous study has investigated the effect of commonly used storage conditions on the preservation of purified α_2_M in its native conformation.

**Table 1:** Examples of conformational dependent α_2_M activities. Many of the activities of α_2_M are dependent on whether or not the protein is in its native conformation or in its transformed state. Additionally, dissociation of the native α_2_M tetramer into dimers (that can be induced using several different chemical methods) has also been demonstrated to influence the activities of α_2_M.

Function	Native α_2_M	Transformed α_2_M	Dissociated α_2_M dimer	References
Protease trapping	Yes	No	No*	[21, 66, 67]
Binding to LRP	No	Yes	Yes, providing the treatment does not denature the receptor binding domain* †	[2, 3, 68]
Chaperone activity	Yes	Yes, α_2_M-protease complexes can also prevent protein aggregation by degrading substrates	Yes, enhanced compared to the native α_2_M* ‡	[13, 16, 17, 32, 69]
Binding to Aβ_1–40_ or Aβ_1–42_ peptide	Yes, but to oligomers only, early on the amyloid forming pathway	Yes, binds monomeric and oligomeric Aβ	Yes, binds monomeric and oligomeric Aβ with higher affinity than native α_2_M*	[17, 32, 45]
Binding to TGF-β1	Yes; K_D_ = 330 ± 130 nM	Yes; K_D_ = 80 ± 11 nM	Markedly reduced compared to native α_2_M*	[40, 70]
Binding to TGF-β2	Yes; K_D_ = 11 ± 3 nM	Yes; K_D_ = 13 ± 2 nM	Markedly reduced compared to native α_2_M*	[40, 70]
Binding to TNF-α	Only weakly; K_D_ > 1.27 ± 0.17 µM	Only weakly; K_D_ > 0.75 ± 0.10 µM	Markedly increased compared to native α_2_M*	[40, 70]

* α_2_M dimers generated by hypochlorite treatment.

† α_2_M dimers generated by thiocyanate treatment.

‡ α_2_M dimers generated by SDS treatment.

Interestingly, there are significant differences between the reported binding affinities of α_2_M to a range of ligands [17, 32, 39–45] and also between the reported efficacies of α_2_M at inhibiting protein aggregation [13, 32]. Given the current limited understanding of the cold-induced conformational changes of native α_2_M, we have investigated the effect of different buffers and storage conditions on the physical properties of α_2_M and its key activities. This has enabled us to identify a suitable method for the long-term preservation of α_2_M in its native state.

## Materials and Methods

Ethical approval for human blood collection was obtained from the Human Ethics Committee at the University of Wollongong (HE02/080). All donors gave their consent in writing prior to blood collection. All chemicals were obtained from Sigma-Aldrich (Castle Hill NSW, Australia) unless otherwise stated.

### Purification of α_2_M

Human blood was obtained from healthy consenting volunteers and supplemented with 0.4 mg/mL sodium heparin. The cells were pelleted by centrifugation (1000 *x g*, 30 min, 4°C) and the blood plasma was collected and further supplemented with EDTA-free Complete Protease Inhibitor Cocktail (Roche Diagnostics Ltd., Castle Hill, Australia), according to the manufacturer’s directions. α_2_M purification procedures were carried out immediately (i.e. the plasma was not stored or frozen) [46]. 5M NaCl and 1M HEPES buffers, pH 7.2, were added to the plasma to achieve final concentrations of 1 M and 20 mM, respectively, and the plasma was filtered through a 0.22 μm membrane. A HiTrap chelating column (GE Healthcare, Silverwater, Australia) was stripped (stripping buffer; 0.5 mM NaCl, 50 mM EDTA, 20 mM HEPES, pH 7.2), washed using milliQ water, recharged (recharge buffer; 0.1 M ZnSO_4_), and then equilibrated in binding buffer (1 M NaCl, 20 mM HEPES, pH 7.2). The filtered plasma was loaded onto the column and unbound proteins were removed by extensively washing with binding buffer. Loosely bound proteins were eluted from the column by washing with 20 mM imidazole, 0.5 M NaCl, 20 mM HEPES, pH 7.2, and discarded. The remaining bound protein was eluted with 500 mM imidazole, 0.5 M NaCl, 20 mM HEPES, pH 7.2, and dialyzed against phosphate buffered saline (PBS; 137 mM NaCl, 2.7 mM KCl, 1.5 mM KH_2_PO_4_, 8 mM Na_2_HPO_4,_ pH 7.4) supplemented with 0.02% (w/v) sodium azide (PBS/Az). The dialyzed protein (2 ml) was fractionated by gel filtration using a HiPrep 26/60 Sephacryl S-300 gel filtration column (GE Healthcare, bed volume 320 mL) and fractions containing purified α_2_M were pooled and stored at 4°C. To test the effect of different buffer formulations, α_2_M was extensively dialyzed against 20 mM phosphate buffer, pH 7.4 or 20 mM Tris, pH 8.0, supplemented with NaCl, sodium azide, sucrose or glycine as described in the text. Selected samples were stored at -20°C with or without prior rapid freezing in liquid nitrogen. To quantify the amount of total protein present in solution, a standard bicinchoninic acid (BCA) assay was used [47]. Where indicated, α_2_M was lyophilized using an Alpha 1–2 LD plus freeze dryer (Martin Christ GmbH, Osterode am Harz, Germany).

### Native polyacrylamide gel electrophoresis (PAGE)

Proteins were diluted in sample buffer (100 mM Tris, 10% (v/v) glycerol, 0.0025% (w/v) bromophenol blue, pH 8.6) and subjected to native PAGE using NuPAGE Novex 3–8% Tris-acetate gels (Life Technologies, Mulgrave, Australia) and Tris-glycine running buffer (25 mM Tris, 192 mM glycine, pH 8.3), according to the manufacturer’s instructions. Gels were stained using InstantBlue (Stratech Scientific, Sydney, Australia). To determine the relative proportions of different proteins present in a sample, the density of the relevant bands on native PAGE were measured using ImageJ software.

### Trypsin binding assay

Trypsin activity was measured using a *N*_α_-benzoyl-L-arginine 4-nitroanilide hydrochloride (BAPNA) assay [48]. A stock solution of bovine trypsin (38 μM) was prepared in 1 mM HCl. Trypsin (3.8 μM) and α_2_M (70 nM) were co-incubated in 50 mM Tris, pH 8.0 containing 5 μM CaCl_2_ (10 min, room temperature (RT)) before soybean trypsin inhibitor (10 μM) was added to the solution. After a further incubation (10 min, RT) 2.5 mM BAPNA was added to the solution and the conversion of BAPNA to p-nitroaniline at 37^°^C was measured at 405 nm using a POLARstar Omega plate reader (BMG Labtech Ltd., Mornington, Australia).

### 4,4′-Dianilino-1,1′-binaphthyl-5,5′-disulfonic acid (bisANS) assay

α_2_M (170 nM) was incubated, in the absence of ambient light, with bisANS (10 μM; 5 min, RT) before the fluorescence was measured using a POLARstar Omega plate reader using excitation and emission wavelengths of 360 ± 10 and 490 ± 10 nm, respectively.

### Protein aggregation assays

Creatine phosphokinase (CPK; 5 μM) was incubated in the presence or absence of α _2_M (340 nM) in PBS and heated at 43°C in a FLUOstar OPTIMA platereader (BMG Labtech Ltd.). The turbidity was continuously measured as absorbance at 595 nm.

### Tissue culture experiments

SH-SY5Y cells corresponding to a human neuroblastoma cell line, were cultured in DMEM:F-12 (Life Technologies) supplemented with 10% (v/v) fetal bovine serum (Bovogen Biologicals, Keilor East, Australia) and maintained in an incubator at 37°C with humidified air containing 5% (v/v) CO_2_. The cells were routinely passaged using trypsin/EDTA (1:250, pH 7.0; Life Technologies). Prior to flow cytometry experiments, SH-SY5Y cells were cultured for 48 hr without passage before being detached using 5 mM EDTA in PBS.

### Flow cytometry analysis

Receptor-associated protein (RAP) inhibits the binding of ligands, including α_2_M, to lipoprotein receptors [49]. The recombinant fusion protein glutathione-S-transferase-RAP was purified as previously described [50]. SH-SY5Y cells were detached (as described above) and washed in Hank’s binding buffer (HBB; 137 mM NaCl, 5.4 mM KCl, 0.34 mM Na_2_HPO_4_, 0.44 mM KH_2_PO_4_, 2 mM CaCl_2_, 2 mM MgCl_2_, 0.1% (w/v) BSA, pH 7.4) by centrifugation. The cells were then incubated step-wise with the following reagents diluted in HBB (all incubations were for 30 min at 4°C and were separated by centrifugal washing of the cells): (i) RAP (8 μM) or HBB alone; (ii) α_2_M (100 nM); (iii) rabbit anti-α_2_M antibody (diluted 1:1000; Dako, North Sydney, Australia), and finally (iv) anti-rabbit IgG-FITC antibody (diluted 1:1000; Chemicon, Boronia, Australia). After the final incubation step, the cells were washed twice before analysis using an LSR-II flow cytometer (Beckton Dickinson, North Ryde, Australia). Viable cells were gated based on propidium iodide exclusion (PI; 3 μM). The acquired data were analyzed using FlowJo7 software (TreeStar Inc., Ashland, OR, USA). Background fluorescence was measured using cells treated as above except without incubation with α_2_M.

### Circular dichroism (CD) spectroscopy

CD experiments were performed using a Jasco J-810 spectropolarimeter (JASCO (UK) Ltd, Great Dunmow UK) equipped with a Peltier temperature controller. α_2_M (270 nM) in sodium phosphate buffer (20 mM, pH 7.4) was analyzed using a 0.1 cm path-length cuvette. For secondary structure analysis, five spectra of each protein sample and of phosphate buffer were recorded between 200 and 250 nm at 20°C (using a scan speed of 50 nm/min with a 1 nm band width and a 4 s response time). The spectra of the samples were averaged and corrected for the signal generated by the buffer alone. In between measurements, the α_2_M sample was removed from the cuvette and flash frozen in liquid nitrogen. The sample was then thawed, briefly centrifuged and re-analyzed. This sequence was repeated five times and the results were compared to ANS binding measurements of matched α_2_M samples.

## Results and Discussion

### Effects of freezing and lyophilization on α_2_M in PBS/Az

**Fig 1:**
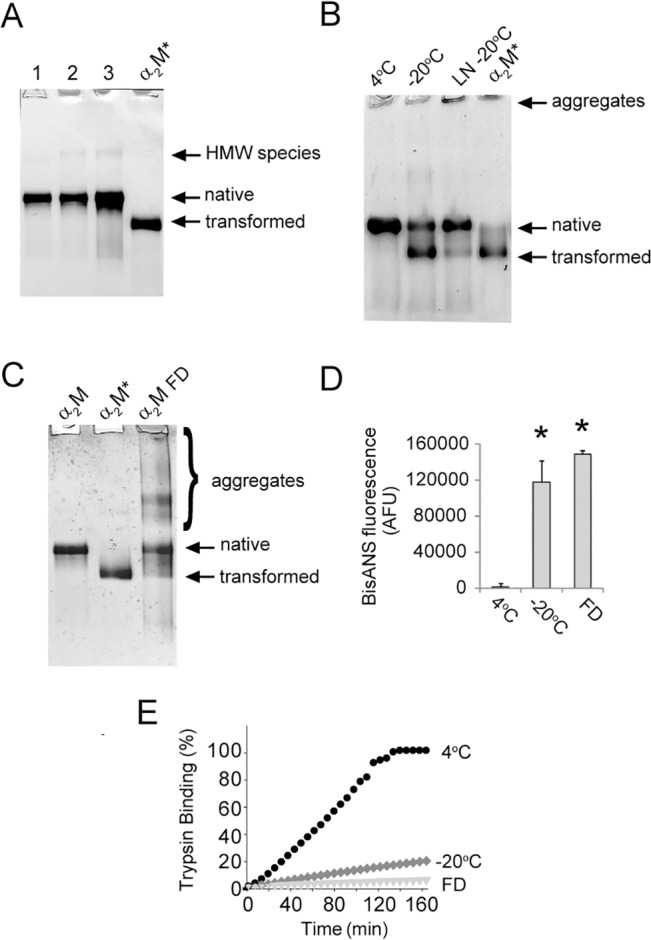
The effects of storage temperature on the conformation of purified α_2_M in PBS/Az. Images of native PAGE (3–8% Tris-acetate) gels showing α_**2**_M (A) freshly purified in PBS/Az (lane 1), after storage in PBS/Az (4°C, 4 months) (lane 2) and after storage in PBS/Az (4°C, 8 months) (lane 3). In all samples, there is a small amount of higher molecular weight (HMW) species present; (B) in PBS/Az (4°C or -20°C, 10 days). Also shown is α_**2**_M in PBS/Az after rapid freezing in liquid nitrogen (LN) and subsequent storage (-20°C, 10 days), and (C) in PBS/Az (4°C, 2 months) or after freeze-drying (FD) and storage (-20°C, 10 days). In images (A-C) the position of transformed α_**2**_M (α_**2**_M*; generated by treatment with 400 mM NH_**4**_Cl in PBS overnight) is shown. (D) bisANS fluorescence measurements for α_**2**_M in PBS/Az (4°C, -20°C, or freeze dried and stored at -20°C, all for 10 days). The results shown are the mean bisANS fluorescence (n = 3±SD) in arbitrary fluorescence units (AFU). (E) Trypsin activity assay showing the conversion of BAPNA to p-nitroaniline by trypsin-α_**2**_M complexes. For this assay α_**2**_M was stored as described in (D). * Denotes significant increases in bisANS fluorescence as a result of storing native α_**2**_M at -20°C, or FD compared to a matched α_**2**_M sample stored at 4°C (Student’s t-test p < 0.01).

α_2_M was purified from human blood plasma under non-reducing conditions using a combination of immobilized metal affinity chromatography (IMAC) and size exclusion chromatography. Using this non-denaturing procedure, more than 20 mg of purified native α_2_M was obtained from 100 ml of blood (the migration of freshly purified α_2_M in native PAGE is shown in Fig 1A, lane 1). When stored in PBS/Az at 4°C, purified α_2_M was found to retain its native conformation for 4 months as assessed by its migration on native PAGE (Fig 1A, lane 2). However, after 8 months of storage under the same conditions, purified α_2_M migrated as a much broader band (Fig 1A, lane 3), suggesting that during prolonged storage some of the protein molecules had altered in structure. Indeed, some molecules appeared to have adopted a more compact structure (migrating slightly faster than the native α_2_M band), while others had formed smaller species (based on the relative mobility of these species they are likely to be dimers, lower band in lane 3) and still others had formed higher molecular weight species (due to non-covalent self-association of the protein). As a commonly used strategy for long-term storage of proteins involves freezing [51], the protein was analyzed after storage in PBS/Az at -20°C for 10 days; less than 50% of the purified α_2_M was found to migrate to a position corresponding to native α_2_M when assessed by native PAGE, the remainder of the protein migrated to a position corresponding to transformed α_2_M (Fig 1B). The extent of α_2_M transformation was found to be reduced, but not abolished, by rapid freezing of the protein in liquid nitrogen (-196°C) prior to storage at -20°C (Fig 1B). Rapid freezing of α_2_M, however, also produced very high molecular weight species that did not readily enter the gel. Analysis of α_2_M by native PAGE after rapid freezing in liquid nitrogen followed by lyophilization from PBS/Az revealed that a heterogeneous mixture of species was present, which migrated at positions corresponding to native, transformed and aggregated α_2_M (Fig 1C). Again, under these conditions, less than 50% of the total protein migrated to the same position as native α_2_M on native PAGE. Also consistent with a loss of native conformation, bisANS fluorescence measurements indicated that when α_2_M was stored at -20°C or lyophilized it had a markedly increased surface hydrophobicity compared to batch-matched α_2_M stored in PBS/Az at 4°C (Fig 1D). Lastly, the trypsin binding activity of α_2_M was reduced by more than 75% after storage at -20°C for 10 days and it was virtually abolished after lyophilization from PBS/Az (Fig 1E). Taken together, the results of these experiments suggest that when stored in PBS/Az at 4°C, α_2_M retains its native conformation for a period of several months, but that substantial losses of native conformation are incurred during freezing and lyophilization procedures.

### Effects of NaCl on the conformation of frozen α_2_M

**Fig 2:**
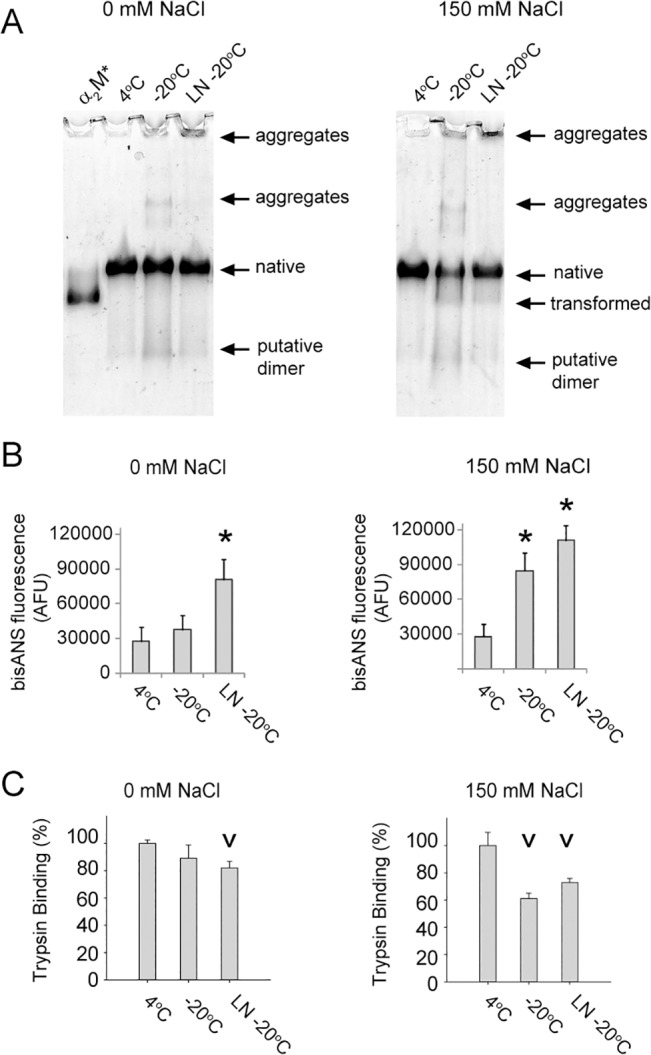
The effect of NaCl on frozen α_2_M preparations. (A) Images of native PAGE (3–8% Tris-acetate) gels showing α_**2**_M stored in 20 mM sodium phosphate buffer, pH 7.4, in the presence or absence of 150 mM NaCl (4°C or -20°C, 20 days). LN indicates that the sample was rapidly frozen in liquid nitrogen prior to storage at -20°C. Also shown is the position of α_**2**_M* (generated by treatment with 400 mM NH_**4**_Cl in PBS overnight). (B) Corresponding bisANS fluorescence measurements for α_**2**_M as described in (A). The results shown are the mean bisANS fluorescence (n = 3±SD) in AFU. (C) Trypsin activity assay showing the rate of BAPNA conversion to p-nitroaniline by trypsin-α_**2**_M complexes generated using α_**2**_M as described in (A). The results shown are the mean BAPNA conversion rates (n = 3±SD). * Denotes significant increases in bisANS fluorescence of α_**2**_M stored at -20°C compared to a matched sample stored at 4°C. ^v^ Denotes significant decreases in the rate of BAPNA conversion to p-nitroaniline by trypsin-α_**2**_M complexes generated using α_**2**_M stored at -20°C compared to a matched sample stored at 4°C (both Student’s t-test p < 0.01).

During freezing of a solvent, salts such as NaCl are partitioned to the aqueous phase and, therefore, markedly increase in concentration. Partitioning of salts and other buffer components in this manner can dramatically alter the solution pH and destabilize proteins [52]. Many biological studies are done in PBS, which contains a physiologically relevant concentration of NaCl (150 mM), and we therefore tested whether or not NaCl was a major factor promoting changes to native α_2_M upon freezing. We prepared matched samples of native α_2_M in 20 mM phosphate buffer, pH 7.4, containing 0 or 150 mM NaCl, and analyzed these by native PAGE following storage for 20 days at -20^°^C. The results suggested that the presence of NaCl produced a small increase (less than 5%) in the proportion of α_2_M transformed under these conditions (Fig 2A). This effect was marginal compared to that observed when α_2_M was stored in PBS/Az at -20°C, which resulted in around 50% of α_2_M being in the transformed state after just 10 days (Fig 1C). In a separate experiment, we confirmed that sodium azide, often used as a biocide to prevent bacterial growth in samples, promoted the transformation and aggregation of α_2_M during storage at -20°C, but not during storage at 4°C for several months (data not shown).

Storage of α_2_M for 20 days in the absence or presence of NaCl at -20°C resulted in a small degree of dissociation of α_2_M as well as some aggregation (the combined visible changes were less than 5%; Fig 2A). Although the quantities of smaller species and aggregates visible by native PAGE were similar between the two samples, the amount of native α_2_M remaining in the sample supplemented with NaCl was reduced by around 50%. Visual inspection of this latter sample revealed that some of the protein had precipitated from solution. Regardless of whether or not NaCl was present, the extent of aggregation and dissociation of α_2_M in phosphate buffer was reduced by rapidly freezing α_2_M using liquid nitrogen prior to storage at -20°C, although, as previously observed in this study, this process generated some very high molecular weight species that were retained in the wells of the gel (Fig 2A). After 20 days of storage at -20°C in the presence of NaCl, the surface hydrophobicity of α_2_M was significantly increased compared to that of a matched sample stored at 4°C as measured by bisANS fluorescence (Fig 2B). This increase did not occur, however, when the α_2_M was stored under the same conditions in the absence of NaCl (despite some visible changes in the migration of α_2_M on native PAGE; Fig 2B). Rapid freezing of α_2_M (which appears to protect against aggregation and transformation that is visible by native PAGE analysis in both the presence and absence of NaCl) did not protect α_2_M preparations from increases in surface hydrophobicity during storage at -20°C (Fig 2B).

**Fig 3:**
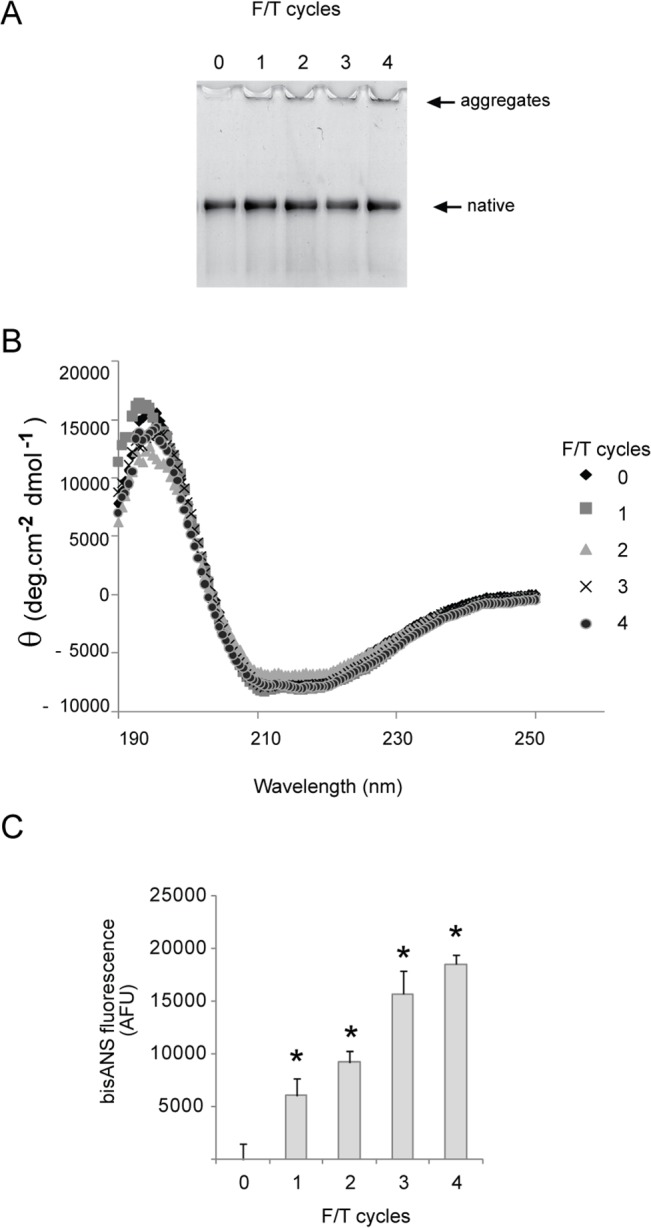
The effect of freezing and thawing on the structure and surface hydrophobicity of α_2_M. (A) Image of a native PAGE (3–8% Tris-acetate) gel showing the migration of α_**2**_M in 20mM sodium phosphate buffer, pH 7.4 after 0–4 cycles of rapid freezing in liquid nitrogen followed by thawing at 37°C (F/T cycles). (B) CD spectra of α_**2**_M as described in (A). (C) BisANS fluorescence measurements (excitation 360 nm, emission 490 nm) for α_**2**_M as described in (A). * Denotes significant increases or decreases in soluble α_**2**_M, bisANS fluorescence or trypsin binding compared to a matched α_**2**_M sample stored at 4°C (Student’s t-test p < 0.01).

In the absence of NaCl and without rapid freezing in liquid nitrogen, native α_2_M appeared to tolerate storage in 20 mM phosphate buffer, pH 7.4 at -20°C, for 20 days; however, after prolonged storage under the same conditions the surface hydrophobicity of the preparation increased 8-fold (Fig 2B). All α_2_M preparations with increased surface hydrophobicity also had significantly decreased trypsin binding activity, consistent with the protein losing its native conformation during storage (Fig 2C). Several cycles of rapid freezing and thawing in phosphate buffer did not greatly alter the migration of α_2_M on native PAGE or induce change to its secondary structure as assessed by CD spectroscopy (Fig 3A and 3B). Despite the minimal variation between the samples when assessed by native PAGE analysis and CD spectroscopy, bisANS fluorescence measurements indicated that exposed hydrophobicity was increased following rapid freezing and thawing (Fig 3C). Therefore, the results show that it is possible to generate α_2_M preparations with markedly different hydrophobicity that are largely indistinguishable by native PAGE analysis.

### Effects of freezing-induced conformational changes on α_2_M interactions with mammalian cells

**Fig 4:**
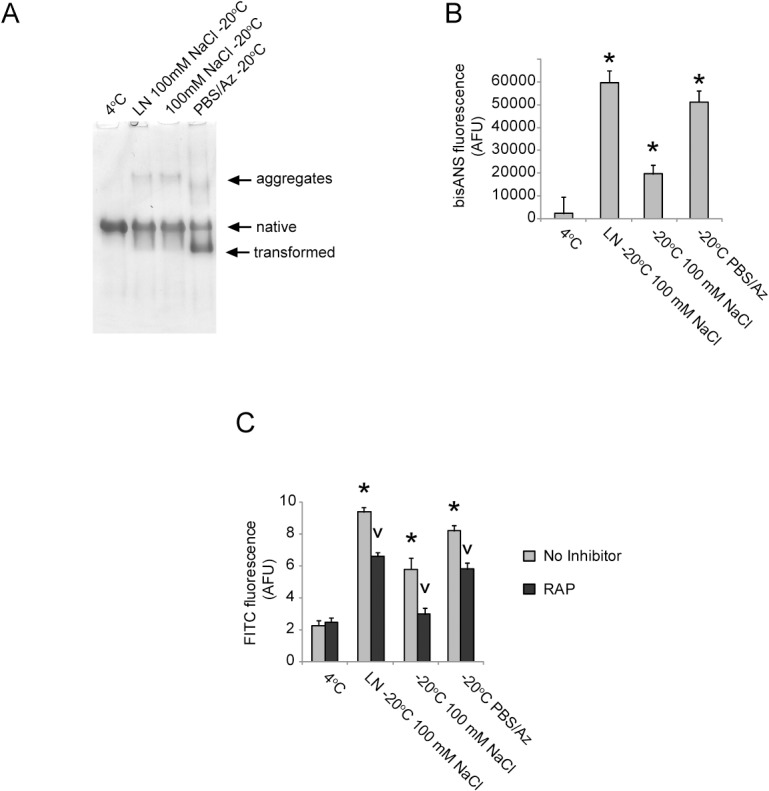
The effect of storage at -20°C on the ability of α_2_M to bind to SH-SY5Y cells. (A) Image of a native PAGE (3–8% Tris-acetate) gel showing α_**2**_M stored in 20 mM sodium phosphate buffer, pH 7.4 containing 100 mM NaCl or in PBS/Az (4°C or -20°C, 10 days). LN indicates that the sample was rapidly frozen in liquid nitrogen prior to storage at -20°C. (B) Corresponding bisANS fluorescence measurements for α_**2**_M stored as described in (A). The results shown are the mean values of bisANS fluorescence (n = 3±SD) in AFU. (C) Flow cytometry analysis showing the binding of α_**2**_M preparations stored as described in (A) to SH-SY5Y cells. The results shown are the composite geometric mean values of FITC fluorescence for 5000 viable cells (n = 3 ± SD) in AFU and are adjusted for background fluorescence. * Denotes significant increases in cell surface binding of α_**2**_M stored at -20°C compared to a batch matched sample stored at 4°C. v Denotes significant decreases in cell surface binding of α_**2**_M as a result of pre-incubation of the cells with RAP.

Transformation of the native α_2_M tetramer into its compact form, or in some cases its dissociation into dimers, reveals a cryptic binding site for the endocytic receptor LRP (Table 1). To examine the effect of changes induced by freezing on the binding of α_2_M to SH-SY5Y cells which express LRP [4], we used a range of storage conditions to generate α_2_M preparations that contained different proportions of transformed α_2_M (assessed by native PAGE) and different degrees of surface hydrophobicity (assessed by bisANS fluorescence measurements) (Fig 4A and 4B, respectively). The results show that the total cell surface binding of α_2_M corresponds more closely with the surface hydrophobicity of the preparation than with the degree of transformation (Fig 4C).

Approximately 20–50% of the cell surface binding of the frozen α_2_M preparations was inhibited by pre-incubating cells with the lipoprotein receptor inhibitor RAP. Surprisingly, the level of RAP-inhibited α_2_M binding did not closely correspond with the quantity of transformed α_2_M detected by native PAGE. This finding was clearly demonstrated by comparing the extent of binding inhibited by RAP of α_2_M stored in PBS/Az at -20°C (which induced ca. 50% of the α_2_M to migrate on native PAGE similarly to transformed α_2_M) to that of α_2_M stored in phosphate buffer supplemented with 100 mM NaCl at -20°C after rapid freezing in liquid nitrogen (which induced less than 5% of the α_2_M to migrate on native PAGE similar to transformed α_2_M). In both cases, ca. 20% of the cell surface binding of the α_2_M appeared to be attributable to lipoprotein receptors. Collectively, the results suggest that loss of the native α_2_M conformation resulting from storage at -20°C increases its binding to the cell surface via both lipoprotein receptor-dependent and lipoprotein receptor-independent mechanisms (e.g. direct binding to the cell membrane or binding to alternative receptors). The precise mechanisms responsible for the lipoprotein receptor-independent binding of α_2_M following freezing are unknown; these may, however, include interactions that are mediated by hydrophobicity.

### Effects of freezing-induced conformational changes on α_2_M chaperone activity

**Fig 5:**
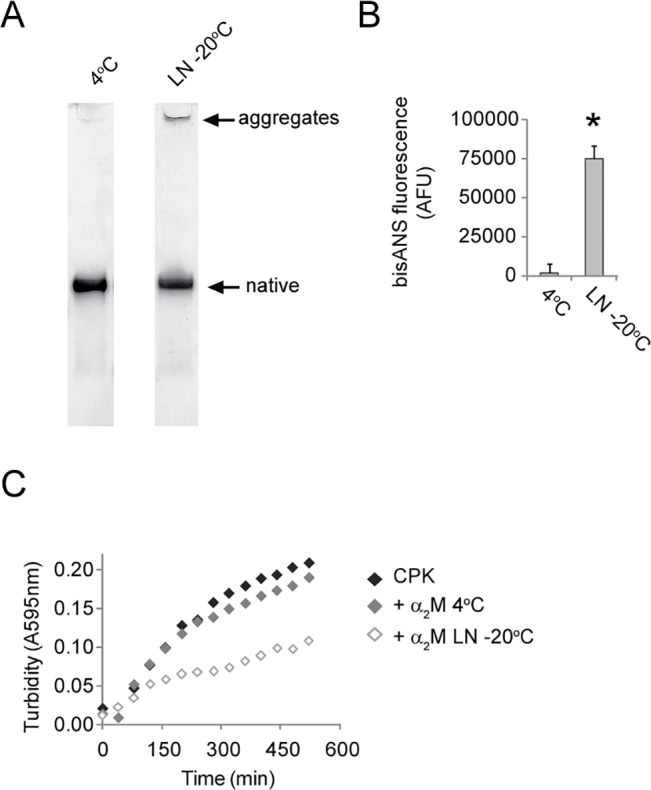
The effect of storage at -20°C on α_2_M chaperone activity. (A) Images of native PAGE (3–8% Tris-acetate) analyses of α_**2**_M stored in 20 mM phosphate buffer, pH 7.4 (4°C or -20°C, 1 month). The latter sample was rapidly frozen in LN prior to storage at -20°C. (B) Corresponding bisANS fluorescence measurements for the α_**2**_M samples described in (A). The results shown are the values of the mean bisANS fluorescence (n = 3±SD) in AFU. * Denotes significantly increased bisANS fluorescence as a result of storage at -20°C (Student’s t-test p < 0.01) (C) Turbidity measurements of CPK aggregation in the presence or absence of α_**2**_M which had been stored at 4°C or -20°C as described in (A). The data are from individual measurements and are representative of several different experiments.

The activity of molecular chaperones is typically dependent on hydrophobic interactions between the chaperone and the client protein [53]. We, therefore, tested the chaperone activity of α_2_M preparations that were largely indistinguishable by native PAGE analysis (Fig 5A), but had significantly different levels of exposed hydrophobic surfaces as assessed by bisANS fluorescence measurements (Fig 5B). Using creatine phosphokinase (CPK), a model client protein that readily aggregates and precipitates when incubated at 43°C, we found that storage at -20°C increased the ability of α_2_M to inhibit the precipitation of CPK (Fig 5C). At a molar ratio of α_2_M-to-CPK of 1:15, α_2_M stored in 20 mM phosphate buffer, pH 7.4 at 4^°^C for 1 month, had virtually no effect on the precipitation of CPK over a period of 8 hrs. In contrast, under the same conditions a matched α_2_M preparation that had been rapidly frozen in liquid nitrogen and then stored at -20°C for 1 month, reduced the precipitation of CPK by ca. 50% (Fig 5C).

### Effects of glycine and sucrose on lyophilized α_2_M

**Fig 6:**
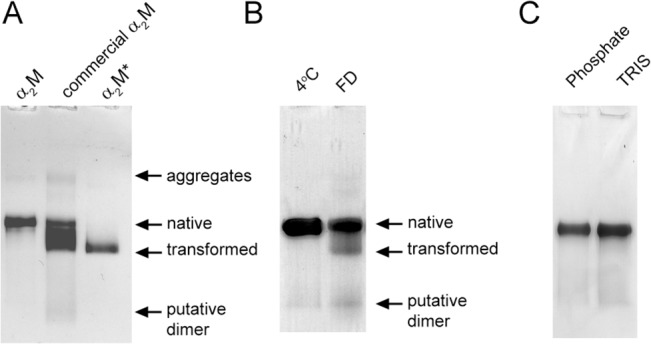
The effect of lyophilization from Tris buffer on purified α_2_M. Images of native PAGE (3–8% Tris-acetate) gels showing the migration of (A) reconstituted α_**2**_M that had been lyophilized from 20 mM Tris, 130 mM glycine, 80 mM trehalose, pH 8.0 and (B) α_**2**_M stored in solution at 4°C in 20 mM Tris, pH 8.0 for 2 months or following reconstitution after it had been lyophilized from 20 mM Tris, pH 8.0 and stored at -20°C for 7 days. As references, the positions of native and transformed α_**2**_M are also shown in (A). (C) Matched α_**2**_M samples in 20 mM phosphate, pH 7.4 or 20 mM Tris, pH 8.0 stored at 4°C for 2 months. Both samples contained 0.02% (w/v) sodium azide.

Many commercial samples of human α_2_M are in a lyophilized form, although the exact formulations used (i.e. buffers, stabilizers and excipients) can vary significantly. We assessed the composition of one such α_2_M preparation lyophilized from 20 mM Tris, 130 mM glycine, 80 mM trehalose pH 8.0, by native PAGE analysis. After reconstitution, the α_2_M was found to contain a heterogeneous mixture of species, mostly corresponding to transformed or partially transformed α_2_M (Fig 6A). Similar analysis of α_2_M purified from fresh human plasma (as described in the Materials and Methods), which was rapidly frozen in liquid nitrogen and lyophilized from 20 mM Tris pH 8.0, supported the conclusion that α_2_M is induced to transform when frozen then lyophilized in Tris buffer (Fig 6B), and that either the stabilizers added to the preparation were insufficient to preserve the native conformation of α_2_M or the α_2_M had been induced to adopt a non-native conformation prior to lyophilization. Similar to native α_2_M stored in 20 mM sodium phosphate, pH 7.4, the native α_2_M stored in 20 mM Tris pH 8.0, appeared to be stable when stored at 4°C for several months (Fig 6C).

**Fig 7:**
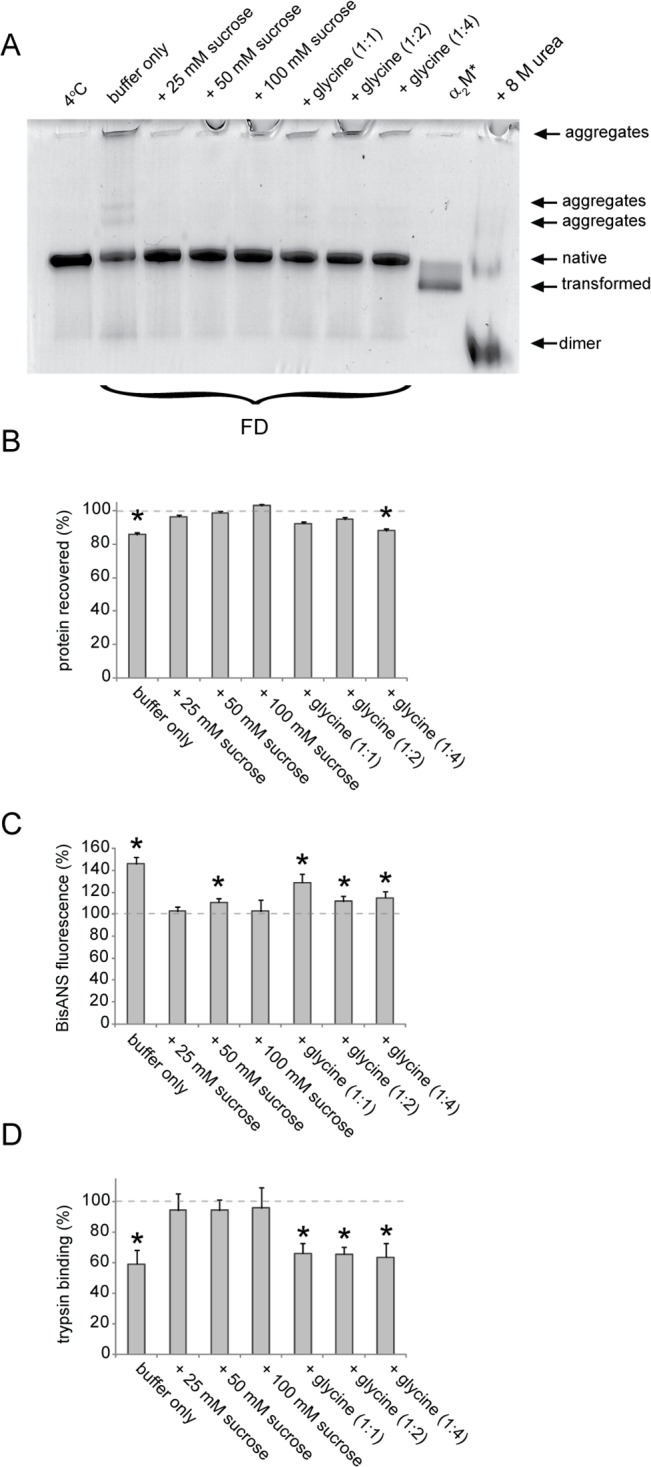
The effect of sucrose or glycine on the preservation of native α_2_M characteristics after lyophilization. (A) Image of a native PAGE (3–8% Tris-acetate) gel showing α_**2**_M stored in 20 mM phosphate buffer, pH 7.4 (4°C, 2 months) or after lyophilization and storage at -20°C for 7 days prior to reconstitution. α_**2**_M was lyophilized from buffer only, with sucrose present at the indicated concentrations, or with glycine at the indicated mass ratios (α_**2**_M-to-glycine). As references, the positions of native α_**2**_M, transformed α_**2**_M and dimeric α_**2**_M (generated by incubation with 8 M urea) are shown. (B) Recovery of soluble α_**2**_M after lyophilization from the conditions described in (A) as assessed by the BCA assay. (C) Corresponding bisANS fluorescence measurements for α_**2**_M after lyophilization from the conditions described in (A). The results shown are the values of the mean bisANS fluorescence (n = 3±SD) in AFU.

In the absence of other buffer constituents (i.e. salt and sodium azide), lyophilization of α_2_M from 20 mM phosphate buffer, pH 7.4, did not transform the protein; however, a fraction of the protein formed higher molecular weight species (consistent with protein self-association) and a further fraction dissociated into smaller species that migrated on native PAGE similarly to urea-dissociated α_2_M dimers (Fig 6A). Given that sucrose and glycine are commonly added as stabilizers for commercially available lyophilized α_2_M preparations, we tested their ability to preserve α_2_M in its native state during freezing and drying [51, 52, 54]. Native PAGE analysis showed that the addition of either sucrose or glycine to α_2_M prior to lyophilization reduced its subsequent aggregation and dissociation; however, when glycine was present some high molecular mass species were still visible in the wells of the gel (Fig 7A). Quantification of the amount of soluble protein recovered after reconstitution of the lyophilized sample using a bicinchoninic acid (BCA) assay indicated 85% recovery of the protein (Fig 7B). The addition of sucrose (25–100 mM) or glycine (at a mass ratio of α_2_M-to-glycine of 1:1 or 1:2) increased the recovery of the lyophilized α_2_M to nearly 100%. In contrast, using a mass ratio of α_2_M:glycine of 1:4 did not significantly improve the recovery of lyophilized α_2_M compare to a matched sample lyophilized from buffer alone (Fig 7B). Consistent with the degree of aggregation assessed by native PAGE analysis, sucrose, but not glycine, preserved the exposed hydrophobicity of the lyophilized α_2_M preparations at the level of matched α_2_M samples stored at 4°C (Fig 7C). As observed for α_2_M preparations stored at -20°C (Fig 2B and 2C), increased hydrophobicity corresponded to decreased trypsin binding activity of lyophilized α_2_M preparations (Fig 7D).

## Conclusion

Despite the fact that functional activities of α_2_M are crucially dependent on its conformation (Table 1), the susceptibility of α_2_M to conformational changes during storage [33, 35–37] has often been overlooked in studies of α_2_M function. The present study, however, indicates that freezing of native α_2_M can result in a loss of protease-trapping activity together with enhanced chaperone activity and increased binding to cells via lipoprotein receptors or by other unidentified mechanisms. All of these effects appear to be associated with an increase in the surface hydrophobicity of α_2_M, which may occur independently of α_2_M adopting the transformed conformation that is known to be more hydrophobic than the native state [55].

We also demonstrate in this study that changes in α_2_M structure that are undetectable by native PAGE analysis can significantly alter its activities. This finding has broad significance because native PAGE analysis is currently the preferred method used to verify the integrity of native α_2_M preparations before conducting functional analyses. Our results strongly suggest that it is not safe to assume that α_2_M is in the native conformation on the basis of native PAGE analysis alone. This conclusion may explain why contradictory results regarding the activities of native α_2_M have been published; particularly in studies involving hydrophobic ligands (see *Introduction*). The current study also enables us to propose a strategy to monitor structural changes of stored α_2_M preparations. This strategy involves the periodic assessment of the surface exposed hydrophobicity of the protein using bisANS fluorescence measurements.

Since their initial characterization in the 1950-60s, bisANS and the related dye 1-anilinonapthalene-8-sulfonate (ANS) have become popular tools for studying surface hydrophobicity and protein aggregation (reviewed in [56]). Both compounds are weakly fluorescent in aqueous solution, but become highly fluorescent when bound to apolar surfaces. It has previously been shown that ANS fluorescence measurements can be used to monitor freezing-induced perturbations of tertiary protein structure with high sensitivity [57, 58]. In the absence of cryoprotectants, an increase in ANS binding induced by freezing is a general property of proteins, but large multi-subunit proteins tend to be more susceptible to destabilization when frozen compared to small monomeric proteins [58]; this latter conclusion is likely to be the result of subunit dissociation. Although the results of this study support that dissociation of α_2_M into dimers may be a contributing factor, it may not be the only structural modification responsible for the increased surface hydrophobicity of frozen or lyophilized α_2_M preparations. Nevertheless, dissociation of non-covalently associated subunits has been demonstrated to influence the chaperone activity of α_2_M and a number of other chaperone proteins [32, 59, 60]; freezing-induced subunit dissociation should, therefore, be closely monitored in chaperone studies.

Prolonged storage of proteins whilst maintaining their physicochemical properties is a complex problem and many investigations about the mechanisms by which proper storage can be achieved have been reported [51, 52, 61–63]. Given the important relationship between structural changes in α_2_M and its functional properties, we have tried to identify, biologically appropriate, storage conditions for this protein. To improve their long-term stability, proteins are commonly stored at or below -20°C, either in solution or in a lyophilized form [51]. Rapid freezing of α_2_M was found to provide some protection against structural changes that were visible by native PAGE analysis (Figs 1C and 2A), although transformation, self-association and dissociation of α_2_M was still observed during prolonged storage at -20°C after rapid freezing. Furthermore, supercooling of α_2_M in liquid nitrogen generated some high molecular weight species with significantly enhanced surface hydrophobicity compared to the native protein (Fig 2A and 2B). Together these data support the conclusion that, in the absence of a suitable cryoprotectant, rapid freezing of α_2_M is not a suitable strategy for preserving its native conformation.

Under the conditions used in this study we found that glycine only marginally improved the solubility of lyophilized α_2_M, and did not preserve its trypsin binding activity (Fig 7). In contrast, the addition of sucrose to α_2_M preparations appears to offer a reliable method for preserving the native structure and activity of α_2_M during prolonged storage in solution at temperatures below -20°C and during lyophilization. During freezing (in solution) sucrose affords cryoprotection by being preferentially excluded from contact with the surface of the proteins, thereby leaving the protein preferentially hydrated [64]; whereas during lyophilization, sucrose prevents damage to proteins by forming hydrogen bonds with the dried protein in place of the lost water [65]. Given that many biological functions of proteins are mediated by hydrophobic interactions, great care must be taken when storing proteins in frozen or lyophilized forms for in vitro studies. We have identified that α_2_M is acutely sensitive to both freezing and lyophilization and that freezing-induced conformational changes significantly influence several of its key activities. While some of these conformational changes result in the visibly altered migration of α_2_M on native PAGE, bisANS fluorescence measurements provide a far more sensitive indication of the degree to which the native conformation of α_2_M has been compromised during storage. Our results, therefore, indicate that, in addition to native PAGE analysis, measurement of the surface hydrophobicity of α_2_M should be adopted as a standard quality control measure for functional studies of this protein.
